# Adapted to Roar: Functional Morphology of Tiger and Lion Vocal Folds

**DOI:** 10.1371/journal.pone.0027029

**Published:** 2011-11-02

**Authors:** Sarah A. Klemuk, Tobias Riede, Edward J. Walsh, Ingo R. Titze

**Affiliations:** 1 Department of Communication Sciences and Disorders, The University of Iowa, Iowa City, Iowa, United States of America; 2 Department of Biology, The University of Utah, Salt Lake City, Utah, United States of America; 3 National Center for Voice and Speech, The University of Utah, Salt Lake City, Utah, United States of America; 4 Boys Town National Research Hospital, Omaha, Nebraska, United States of America; University of Queensland, Australia

## Abstract

Vocal production requires active control of the respiratory system, larynx and vocal tract. Vocal sounds in mammals are produced by flow-induced vocal fold oscillation, which requires vocal fold tissue that can sustain the mechanical stress during phonation. Our understanding of the relationship between morphology and vocal function of vocal folds is very limited. Here we tested the hypothesis that vocal fold morphology and viscoelastic properties allow a prediction of fundamental frequency range of sounds that can be produced, and minimal lung pressure necessary to initiate phonation. We tested the hypothesis in lions and tigers who are well-known for producing low frequency and very loud roaring sounds that expose vocal folds to large stresses. In histological sections, we found that the Panthera vocal fold lamina propria consists of a lateral region with adipocytes embedded in a network of collagen and elastin fibers and hyaluronan. There is also a medial region that contains only fibrous proteins and hyaluronan but no fat cells. Young's moduli range between 10 and 2000 kPa for strains up to 60%. Shear moduli ranged between 0.1 and 2 kPa and differed between layers. Biomechanical and morphological data were used to make predictions of fundamental frequency and subglottal pressure ranges. Such predictions agreed well with measurements from natural phonation and phonation of excised larynges, respectively. We assume that fat shapes Panthera vocal folds into an advantageous geometry for phonation and it protects vocal folds. Its primary function is probably not to increase vocal fold mass as suggested previously. The large square-shaped Panthera vocal fold eases phonation onset and thereby extends the dynamic range of the voice.

## Introduction

Vocal production is a complex behavior during which airflow generated in the respiratory system sets vocal folds in the larynx in motion to generate sound. Producing sound by flow-induced vocal fold oscillation can expose the tissue to large mechanical loads [1]. Vocal fold morphology and viscoelasticity are likely to reflect adaptations to these stresses [2]–[8]. Important acoustical and physiological variables, such as fundamental frequency (F0) and subglottal pressure (Ps), should therefore be predictable from morphology and mechanical properties of vocal fold tissues. Alternatively, if only a generic design is used to produce a wide range of vocal sounds, predictions should be less accurate. This hypothesis was tested in lion and tiger (*Panthera*, *Felidae*) vocal folds. Lions and tigers expose vocal folds to tensile and shear stresses during vocal production [9] producing powerful low-pitched vocalizations. Unlike the vocal fold of most other mammals, Panthera vocal folds contain a layer of fat [10] which provides the opportunity to investigate the function of fat in vocal folds. Fat is used for vocal fold repair in humans (e.g. [11]), but rarely occurs naturally in vocal folds.

Vocal fold viscoelasticity is best quantified in terms of shear properties (rheologic biomechanics) and tensile properties, representing two major types of loading during phonation. When vocal folds oscillate the tissue moves simultaneously along two main axes, medio-lateral and caudo-cranial, causing shear stress. Mammalian vocal folds are also exposed to tensile stress. They are positioned between the arytenoid cartilage and thyroid cartilage, almost like a string between two anchor points. In order to achieve a higher F0, vocal folds are actively elongated to put them into higher tension, causing additional tensile stress [12], [13]. Furthermore, vocal fold tension increases with lung pressure because fibers are elongated at their maximum excursion in large-amplitude vibration [14].

Low frequency and loud roaring sounds are hallmarks of the lion and tiger vocal repertoire. A 36–81 Hz roar of about 0.5 to 1.5 s in duration reaches sound pressure levels of 114 dB SPL at 1 m [15], [16]. A lion's roar is delivered in bouts, lasting up to 90 seconds, consisting of up to 50 calls with F0 of 40 to 200 Hz [16]–[18]. A tiger's roar, often referred to as a ‘long distance advertisement call’, is uttered at an average F0 of 158 Hz (range from 83 Hz to 246 Hz, [19]). Experiments with excised tiger and lion larynges showed vocal fold excursions up to 3 mm at F0 of 150 Hz [9]. The minimum air pressure below the vocal folds to initiate oscillation (phonation threshold pressure), was between 0.2 and 0.6 kPa [9].

Mechanical properties of the vocal folds determine their response to deformation (stretch or shear). They also dictate oscillation threshold pressure (a measure for the ease of oscillation) and fundamental frequency (the most important perceptual acoustic parameter) [13], [20]–[23]. Mechanical properties depend on the tissue design. Mammalian vocal folds consist of epithelium, connective tissue (called *lamina propria*) and thyroarytenoid muscle. The *lamina propria* consists of cellular components (e.g. fibroblasts) and extracellular matrix (elastin, collagen, hyaluronan) in a species-specific organization (e.g. [24]–[27]) contributing to species-specific viscoelastic properties [2]. The lamina propria of Panthera vocal folds also contains large amounts of fat cells [10]. The fat in the Panthera vocal folds has been interpreted as an adaptation to produce low frequency and very loud sounds by increasing vocal fold mass [10]. However, mass is not a good predictor of fundamental frequency [28], whereas length and tissue elasticity are. An alternative and/or additional function of the large volume seems likely.

We report here on three tests. First, the fat distribution in the lamina propria of tiger and lion vocal folds was studied in stained tissue sections. Second, mechanical tests were performed on fat-containing and fat-free vocal fold tissue. Finally, the empirical data were used for simple mathematical predictions of fundamental frequency and phonation threshold pressure. Such predictions are compared with what has been recorded in natural phonation or in excised larynx experiments.

## Materials and Methods

### Specimen

Tissue collection was approved by the Boys Town National Research Hospital Institutional Animal Care and Use Committee (Protocol Numbers 04-04, 07-04 and 10-04). Larynges were harvested from 6 animals euthanized for clinical reasons related to old age by zoo veterinarians at Omaha's Henry Doorly Zoo (Table 1).

**Table 1:** Specimen details. vocal fold length measured between insertion at thyroid cartilage and ventral tip of processus vocalis of arytenoid cartilage.

specimen	sex	age (years)	highest body weight on record (kg)	membraneous vocal fold length (cm)	procedures performed
Lion 1	female	18	158	3.5	histology,
(*Panthera leo*) (Isis No. 5333)					ergometry
Lion 2	female	19	130	4.2	rheology,
*(Panthera leo*) (Isis No. 15825)					ergometry
Lion 3	female	15	178	4.7	rheology
*(Panthera leo*) (Isis No. 15828)					
Sumatran tiger	female	22.4	101	3.7	rheology,
(*Panthera tigris*) (Isis No. 10153)					ergometry
Amur (Siberian) Tiger	male	16.4	182	4.6	histology,
(*Panthera tigris*) (Isis No. 6220)					rheology, ergometry
Bengal Tiger	female	18.4	147	4.1	histology,
(*Panthera tigris*) (Isis No. 5380)					ergometry

Tissue was collected immediately after death, submerged in saline solution, quick frozen in liquid nitrogen and maintained at −80° C until the experiment. The effects of freezing on viscoelastic properties of vocal folds remain within a 5% margin [29], [30]. After slowly thawing a specimen, vocal folds were carefully excised and submitted either to tensile tests or rheology (Table 1). For histological investigations, the vocal folds were not excised but fixed in situ before further dissections.

### Histology

Specimens were fixed in 10% buffered formalin phosphate (SF100-4; Fisher Scientific) for 4 weeks. Mid-membraneous coronal sections (5 µm thick) were stained with haematoxylin-eosin for a general overview, Masson's Trichrome (TRI) for collagen fiber stain, Elastica-Van Gieson (EVG) for elastic fiber stain or alcian blue (AB) stain (pH 2.5) for mucopolysaccharides and glycosaminoglycans. We also performed a digestion procedure with bovine testicular hyaluronidase (2 hours at 37°C) in combination with a subsequent alcian blue stain. Incubation with bovine testicular hyaluronidase increases specificity for various acid mucosubstances in the alcian blue stain. If hyaluronan is a major component of the mucosubstances, alcian blue stain is destroyed. All stains were performed in conjunction with control stains in order to confirm positive stains for the respective material (liver for TRI, artery for EVG; umbilical cord for AB) on human tissue.

Sections were scanned with Imagescope software (v. 8.2.5.1263; Aperio Tech.). The intensity of stains was quantified in Image J (version 1.41o; NIH open source). Images originally saved in RGB mode, were converted to a 3-slice (red, green, blue) stack. The blue channel (TRI, AB) and the red channel (EVG) were further analyzed. A positive pixel count was performed in Image J. Trichrome and EVG stained images were used to determine the relative amounts of collagen and elastin. Alcian Blue stained images before and after hyaluronidase treatment were compared to quantify the difference in the blue intensity before and after hyaluronidase treatment.

### Tensile measurements

Tensile tests were conducted in the same setup detailed in other studies [2], [3], [6], [31]. In short, force-elongation data were obtained by 1 Hz sinusoidal stretch and release of the vocal fold tissue by a servo-control lever system (Model 305B; Aurora Scientific, Aurora, ON, Canada). A 2-cm long segment of vocal fold lamina propria was dissected. A fragment of arytenoid cartilage was retained at one end for mounting. The epithelium was carefully removed. A suture connected the arytenoid cartilage fragment to the lever arm of the servo-control lever system. A clamp held the ventral end of the *lamina propria* in a fixed position. The tissue was vertically mounted in a water-surrounded chamber containing Ringer solution, maintained at 38°C. The exact length between cartilage and fixation point in the clamp was measured with a caliper (+/−0.1 mm accuracy).

Lever arm displacement and force (resolution 1 µm and 0.3 mN) were recorded. Elongation was applied in a longitudinal direction (dorso-ventral) followed by a shortening to the original length. The process of loading and unloading was performed 15 times on each sample. The present set of experiments was conducted with a system under displacement control. The force and elongation signals were then transmitted via a 16-bit analog-to-digital acquisition board (Windaq Model DI722, DATAQ Instruments; Akron, OH) at 400 Hz sampling frequency to a PC. True tensile strain (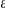) was calculated as a logarithm of specimen length divided by its original mounting length. True tensile stress (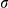) is the ratio between force and cross sectional area. The latter was calculated indirectly using tissue volume and density and assuming isotropy.

A piecewise linear and nonlinear model was used to approximate stress-strain curves as follows:
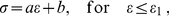
(1)a low strain linear function, where *a* is the slope of the curve and *b* is the y-axis intercept, and
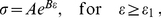
(2)a high-strain exponential function, where *A* and *B* are constants determined empirically.

### Shear viscoelastic measurements

The parallel-plate setup used here, is explained in detail elsewhere [32]–[34]. Briefly, vocal fold tissue was trimmed to make approximate circular tissue samples. Each sample was centered on the base plate of the rotational rheometer (Malvern Instruments, Worcestershire, UK). The upper plate (plate diameters were 38, 30 or 25 mm depending on the size of the sample) was lowered onto the sample. Both base and upper plate were covered with wet/dry sandpaper to reduce sample slippage. Sample thickness was recorded at the gap where the normal force was approximately 50 grams, and the upper plate was not free to rotate. From that position, the gap was reduced to compress the sample by 40–50%. The gap loading criterion [35] was met or exceeded for all materials when gaps were 0.725–1.8 mm. An amplitude sweep was performed at 1 Hz to ensure small amplitude movement occurred. Specimens were moistened with saline throughout testing to avoid drying and subsequent change in rheologic properties. Linear viscoelastic measurements (0.1–100 Hz) were made with the rheometer, following standard rheometry technique. The shear elastic and viscous moduli, *G*′ and *G*″ respectively, are frequency-dependent quantities describing how a material deforms and flows when subjected to forces of small-amplitude [21]. The property of elastic recoil or energy storage is reflected in the *G*′ modulus and the property of flow or energy loss is reflected in the *G*″ modulus. These so-called shear viscoelastic properties were measured because of their defining role in self-sustained oscillation. Their magnitudes are used to quantify the biomechanical-to-aerodynamic energy transduction that occurs in the glottis during vocalization [36].

Loss tangent is the ratio of elastic to viscous moduli (*G*″/*G*′). It is a universal term to describe how fluid-like or solid-like a material is. If the loss tangent approaches 1, viscosity becomes more dominant than elasticity. Error determination of the loss tangent was carried out using the error propagation equation [37]:
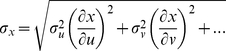
(3)where σ_x_ is the uncertainty of the function of interest *x*, σ_u_ is the standard deviation of parameter *u*, and σ_ν_ is the standard deviation of parameter *v*. We want to understand how errors transfer to a new quantity that is a function of measured parameters, each of which have error. It is assumed that errors of each parameter are independent and relatively small so that second-order terms are negligible. The resulting absolute error for the loss tangent is
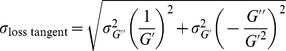
(4)where σ_loss tangent_ is the error in the loss tangent, σ_G′_ is standard deviation of *G*′, and σ_G″_ is standard deviation of *G*″. From this error calculation, the standard error was computed by dividing the σ_loss tangent_ by the square root of the number of samples, just as was done for *G*′ and *G*″.

### Fundamental frequency

The fibrous nature of the vocal fold tissues along the dorso-ventral direction invites comparison of vocal fold oscillation with that of a string. The frequency of vibration (F0) of an ideal string depends primarily on vocal fold length and stress in the fibers,
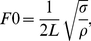
(5)where *L* is the string (or vocal fold) length, *σ* is the tissue stress (tension per unit area, not to be confused with the same symbol used for standard deviation above), and *ρ* is the tissue density (1.02 g/cm^3^). The stress-strain data collected here were used in Eq. 5, and F0 curves for all individuals were calculated.

### Phonation threshold pressure

Phonation threshold pressure, P_th_, is the minimum subglottal pressure required to induce self-sustained vocal fold vibration. It is a measure for the ease of phonation. The measurement of viscoelastic properties transverse to the fibers allow predictions about the phonation threshold pressure [34], [36]. P_th_ was derived mathematically as follows [13], [36]:
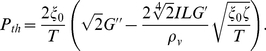
(6)where *G*′ is shear elastic modulus, *G*″ is viscous modulus, *L* is vocal fold length, *T* is vocal fold thickness, *ρ* is tissue density (1020 kg/m^3^), *I* is vocal tract inertance, 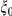 is glottal half width. Owing to tissue incompressibility, increased vocal fold length corresponds to decreased thickness [34]. Vocal tract inertance (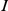) was estimated to be 9.5 kg/m^4^ based on the following relation:
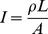
(7)where *L* is the vocal tract length (here only a 5 cm long tube corresponding to the laryngeal vestibule), *A* is the vocal tract cross-sectional area (7 cm^2^), and *ρ* is air density (1 kg/m^3^). Glottal half width values were varied between 0.5 and 4.5 mm.

## Results

### Histology

**Figure 1:**
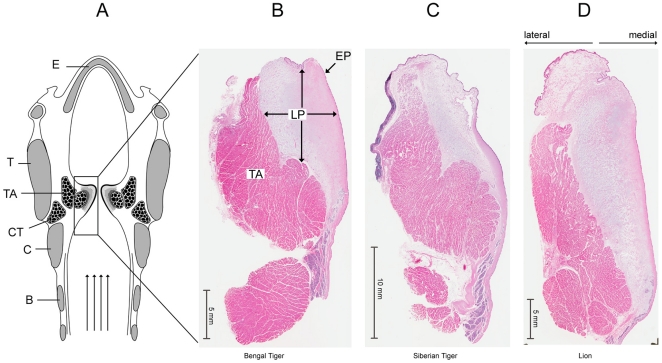
Panthera vocal folds possess a large *lamina propria*. A: Schematic frontal section through a larynx. The square indicates where the histological sections (Haematoxylin-eosin stain) in B, C and D were taken from. The mid-membraneous sections of the vocal fold are from Bengal tiger (**B**), Siberian tiger (**C**), and Lion (**D**). The terms ‘lateral’ and ‘medial’ are used in reference to the body axis and are indicated in D to explain how they apply to the vocal fold. In all three species, the vocal fold consists of epithelium, lamina propria and the thyroarytenoid muscle. LP: lamina propria; E: epiglottis; T: thyroid cartilage; TA: thyroarytenoid muscle; CT: cricothyroid muscle; C: cricoid cartilage; B: tracheal ring. Arrows in A indicate the airflow during expiration. Horizontal arrows in LP of B indicate medio-lateral depth. Vertical arrows in LP of B indicate cranio-caudal thickness.

Vocal folds of lion and tiger specimens are composed of the classical three components of mammalian vocal folds: epithelium, *lamina propria* and thyroarytenoid (TA) muscle (Figure 1). The *lamina propria* ranged in medio-lateral depth between 7.0 mm (Siberian tiger) and 8.4 mm (Lion 1). The cranio-caudal thickness of the *lamina propria* ranged between 21 mm (Siberian tiger) and 34 mm (Lion 1). Arrows in Figure 1B indicate where these measures were taken.

**Figure 2:**
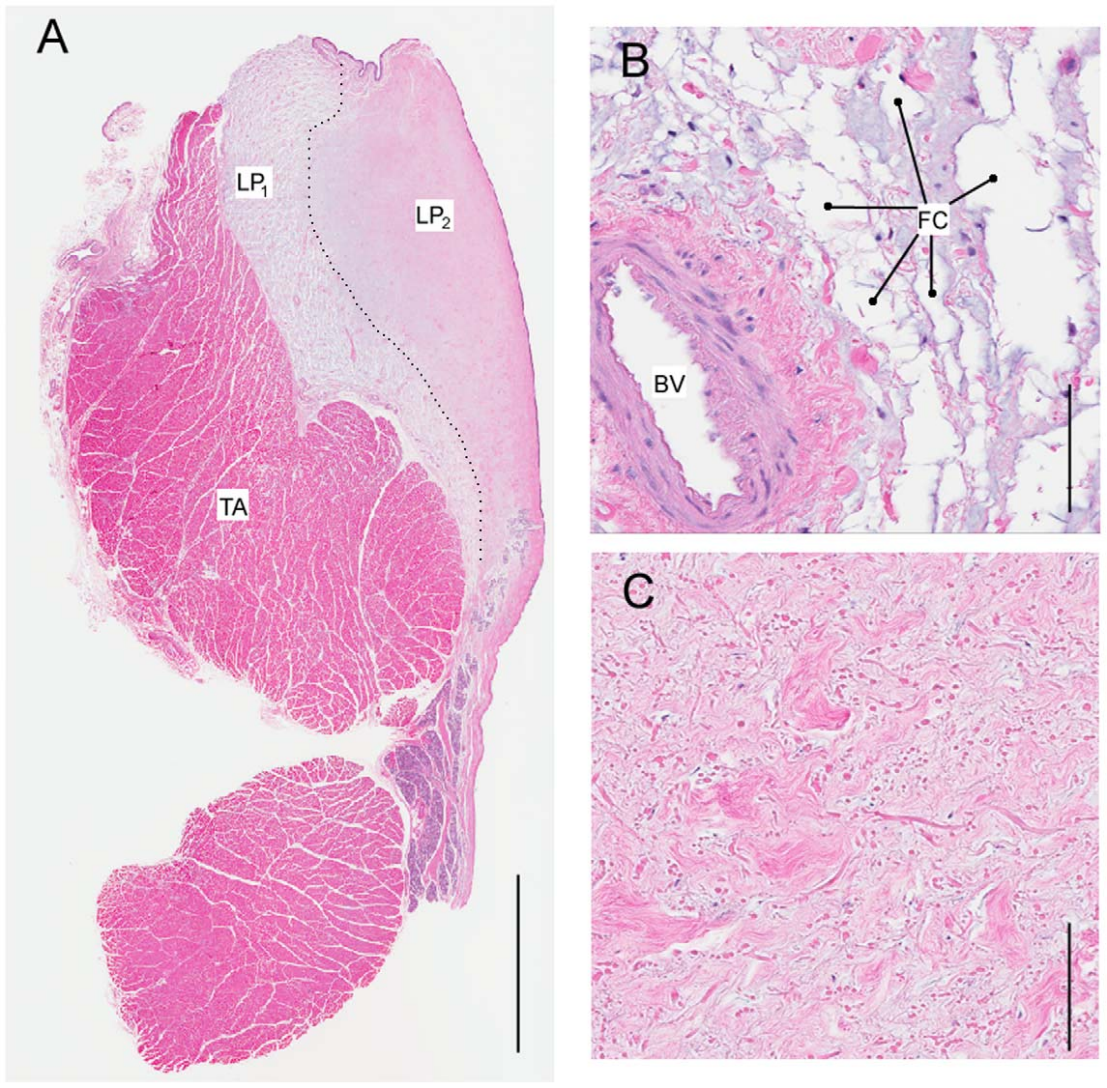
Fat cells are only found in a deep portion of the *lamina propria*. Haematoxylin-eosin stain of a Bengal tiger vocal fold. **A**: Overview of a midmembraneous section indicating a densely packed lamina propria without fat cells medially (LP_2_) and with fat cells laterally (LP_1_). The approximate border between both regions is indicated by a dotted line. Bar indicates a 5 mm distance. Larger magnification of an area in LP_1_ region (**B**) and in LP_2_ region (**C**) are shown. Bars in **B** and **C** indicate a 100 µm distance. TA: thyroarytenoid muscle; LP: lamina propria; BV: blood vessel; FC: fat cells.

**Figure 3:**
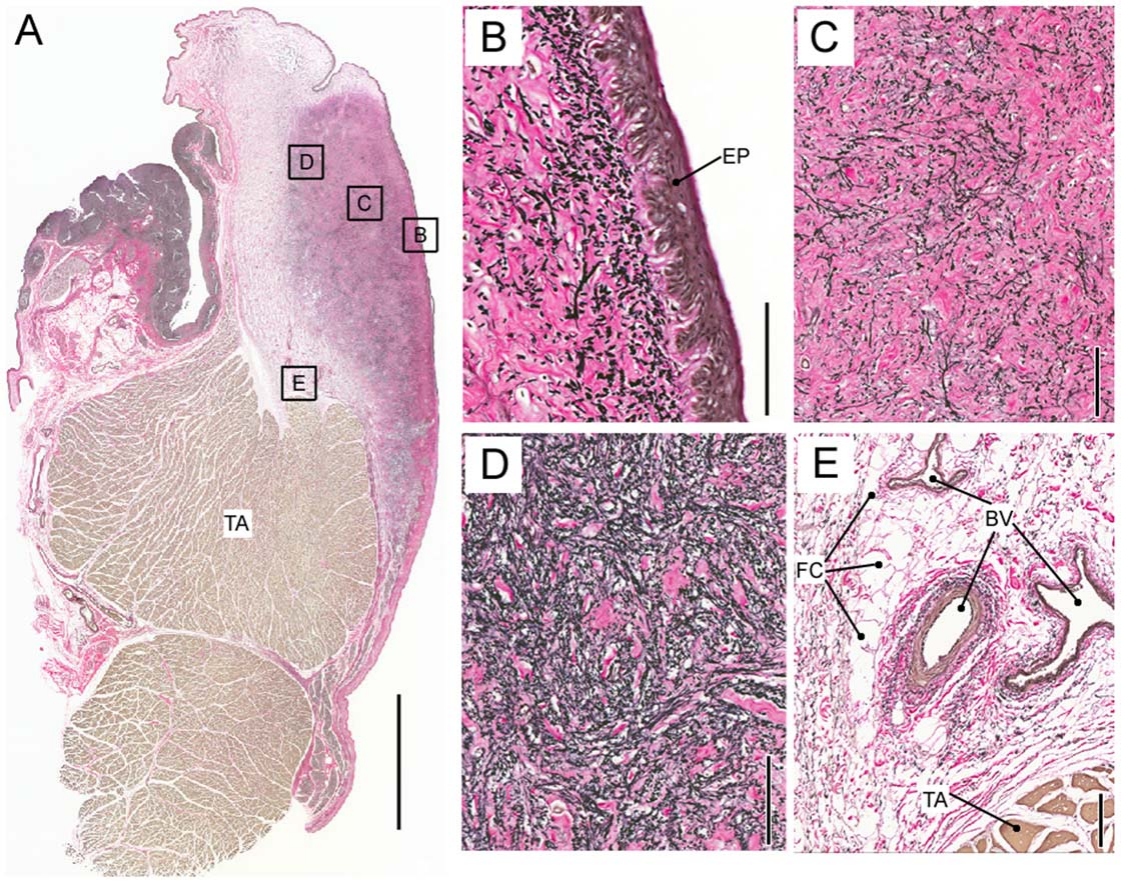
Elastic fibers are densely packed in the medial Lion vocal fold. A: Overview of a midmembraneous section (Elastica-van-Gieson stain). Bar indicates a 5 mm distance. Larger magnifications indicated by four squares in the overview are shown in **B**–**E**. The black dots in **B** are cross sections of elastic fibers. They indicate that most of the elastic fibers are oriented dorso-ventrally. More laterally (**C**, **D** and **E**), fibers are cut along as well as perpendicular to their longitudinal axis suggesting that fibers are more variably oriented. Bars in **B** and **C** indicate a 100 µm distance. TA: thyroarytenoid muscle; BV: blood vessel; FC: fat cells; EP: epithelium.

**Figure 4:**
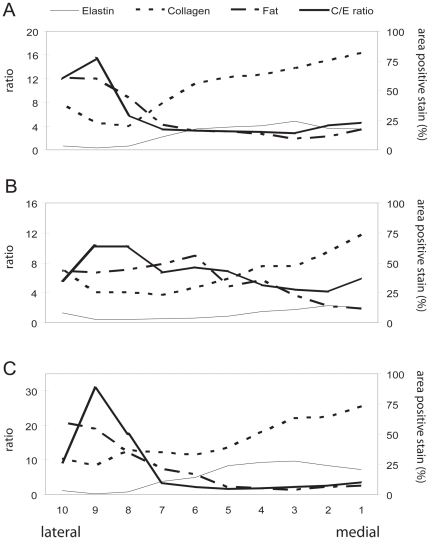
Proteins and fat cells are distributed differently in the medial and lateral *lamina propria*. Elastin, collagen and fat distribution along a medio-lateral transect for Siberian Tiger (**A**) Lion (**B**) and Bengal Tiger (**C**). The area is the relative number of pixels stained positively (elastin, collagen) or representing empty fat cells. See Figure 1 for an explanation of ‘lateral’ and ‘medial’.

The distribution of fat cells in the *lamina propria* is not uniform throughout the *lamina propria*. As shown in Figure 2 for a Bengal tiger, we observed a lateral part of the *lamina propria* that contains fat cells (hereafter labeled “LP1”) and a medial strip of the *lamina propria* that does not contain fat cells (hereafter labeled as “LP2”). Figure 3 shows a similar construct for a female lion. In both tiger specimens and the lion specimen, fat was only found in a deep portion of the *lamina propria*. Figure 4 shows a distribution of fat, elastin, and collagen (lateral to medial) for the three specimens.

**Figure 5:**
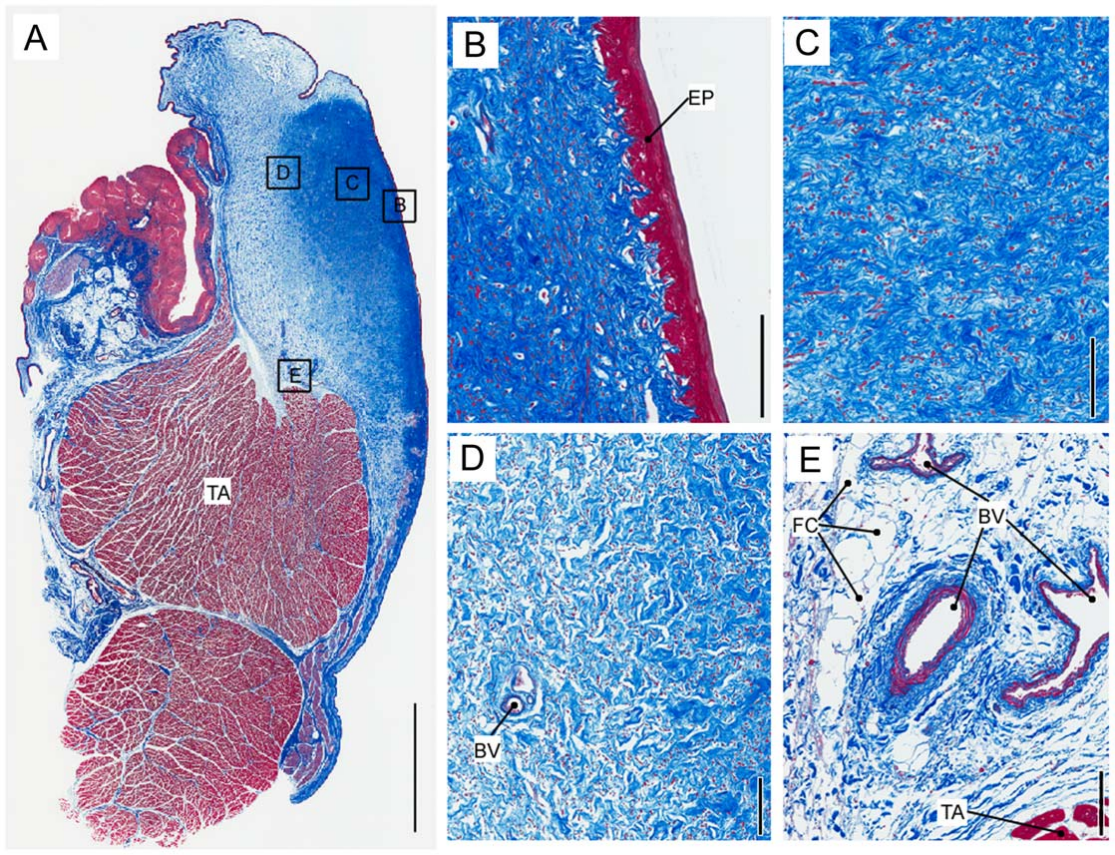
Collagen fibers are densely packed in the medial layer and provide a bed for fat cells in the lateral portion. **A**: Overview of a midmembraneous section (Trichrome stain) of a lion vocal fold. Bar indicates a 5 mm distance. Larger magnifications indicated by four squares in the overview are shown in **B**–**E**. Bars in **B** to **E** indicate a 100 µm distance. TA: thyroarytenoid muscle; BV: blood vessel; FC: fat cells; EP: epithelium.

Elastin and collagen fibers are found throughout the *lamina propria*. Concentrations of both components increase from lateral to medial but change disproportionately (Figure 4). The collagen/elastin ratio indicates elastin is the dominant protein laterally and collagen dominates the fibrillar design medially. Collagen fibers in *Panthera* LP2 do not show preferential directionality. Beneath the epithelium, densely packed collagen fibers make up the majority of the material of the *lamina propria* (Figure 5B). The orientation of elastin varies. Beneath the epithelium we find mostly round cross sections of elastic fibers, suggesting they are dorso-ventrally oriented (Figure 3B). In more lateral regions of the *lamina propria*, elastic fibers were more variably oriented, as evidenced by the observation of cross sectional and longitudinal profiles (Figures 3C–E). The pattern of a densely packed layer medially and a network of fat cells, collagen and elastic fiber more lateral, was found in all three specimens investigated and throughout both layers of the *lamina propria*.

**Figure 6:**
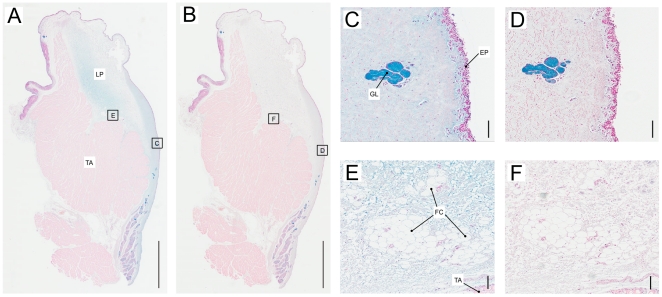
Hyaluronan is found throughout the *lamina propria*. Overview of an alcian blue stain (**A**) and an alcian blue stain after hyaluronidase treatment (**B**) of two consecutive midmembraneous sections of a Siberian tiger vocal fold. Sections in (**C**) to (**F**) are larger magnifications of two different locations of the lamina propria. The locations are indicated by little squares with the respective letter in (**A**) and (**B**). (**C**) and (**D**): locations in the densely packed lamina propria without fat cells medially (LP_2_ in Figure 2) and (**E**) and (**F**) are lamina propria locations with fat cells laterally (LP_1_ in Figure 2). Scale bar in **A** and **B** is 5 mm, scale bar in **C**–**F** is 100 µm. TA: thyroarytenoid muscle; LP: lamina propria; FC: fat cells; GL: glands; EP: epithelium.

The removal of hyaluronan by hyaluronidase digestion with subsequent alcian blue stain indicated that much of the positive stain in the vocal fold can be attributed to hyaluronan (Figures 6A–B). Although the extracellular matrix of vocal folds was mainly made of hyaluronan, which was digested away by hyaluronidase, it was a composition of various mucopolysaccharides and glycosaminoglycans. This pattern was found in all three specimen.

Epithelium thickness ranged between 40 and 62 µm. The deep border of the epithelium is irregular with ‘tongues’ of lamina propria extending up toward the luminal surface. The epithelial protrusions into the *lamina propria* are between 30 and 60 µm deep and were found in lion and tiger vocal folds (Figures 3B, 5B).

The thyroarytenoid muscle is divided into two compartments, occupying a depth of 5–10 mm.

Notable is the large flat medial surface of the vocal fold. This is unlike vocal folds of many other mammals which reach further into the laryngeal lumen more cranially rather than caudally, creating a convergent glottal area. The Panthera vocal fold surfaces are rather flat and parallel from cranial to caudal. Hast [10] described this as a ‘vocal pad’.

### Tensile measurements

**Figure 7:**
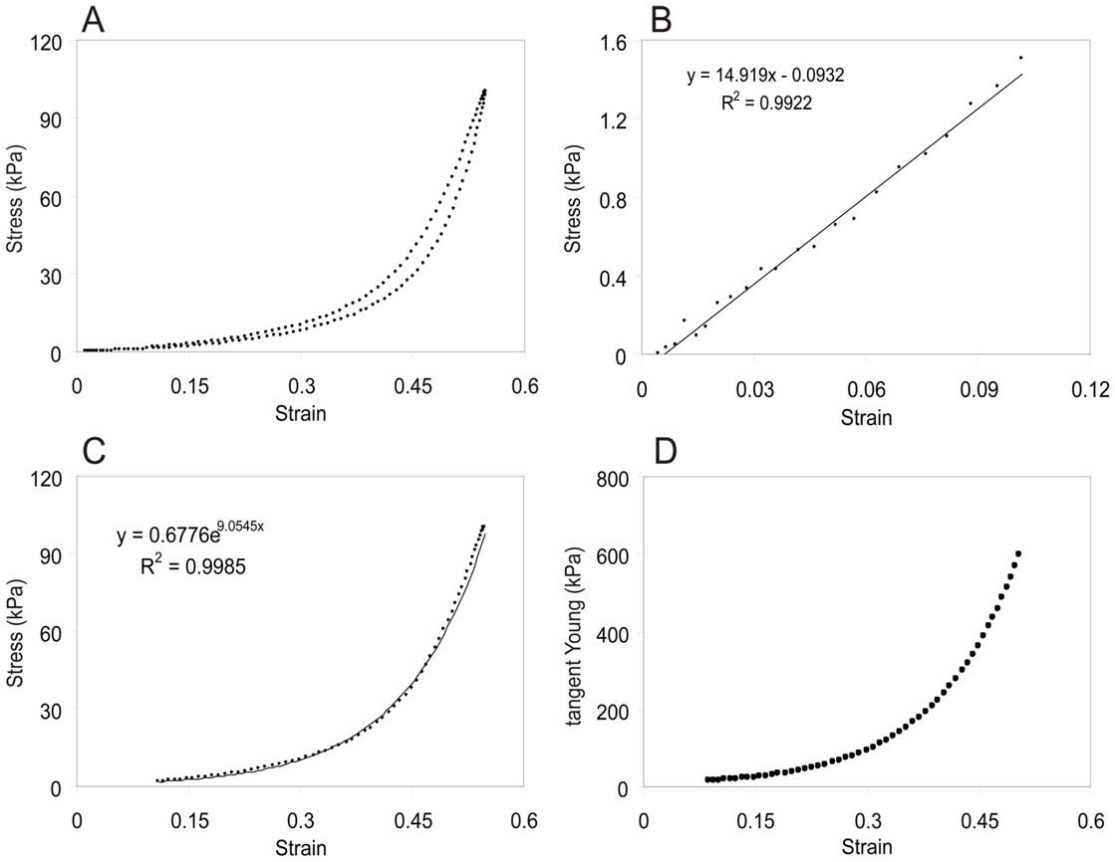
A linear followed by a nonlinear stress response at about 15% strain occurs when Lion 2 vocal fold *lamina propria* is subjected to a 1 Hz sinusoidal elongation. **A**: loading and unloading phase. The upper part of the “banana-shaped” curve is the loading phase (stretching). The lower part is the unloading phase (relaxation). The difference between both curves is due to hysteresis of the tissue, i.e., lower stress in the tissue during the unloading phase. The low strain region of the loading phase was fitted with a linear regression line (**B**), while the high-strain region was modeled with an exponential function (**C**). The limit of the linear region (‘Linear strain limit’) was determined by maximizing the sum of the two regression coefficients (‘sum of r^2^’). **D**: Tangents modulus versus strain for the high-strain (>Linear strain limit) region.

The stress-strain response results in a ‘banana-shaped’ curve (Figure 7A), with a loading part (upper curve) higher than the unloading part (lower curve). This is attributed to viscous energy loss in the cycle (hysteresis). A combination of linear and nonlinear model can best describe the stress-strain curve of the loading curve (Table 2). Regression coefficients were 0.97 and higher (Figure 7, Table 2). The linear strain limit (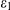) ranged between 7% and 11% strain (Table 2). Young's modulus at 40% strain was 400 kPa on average.

**Table 2:** Parameters of the linear model (Eq. 6) for curve-fitting the empirical stress-strain response of the lamina propria; linear strain limit (ε_1_) in %; and parameters of the exponential model (Eq. 7) for curve-fitting the empirical stress-strain response of the lamina propria.

specimen	a	b	ε_1_	A	B	E (kPa)	F0
Lion 1	10.9	−0.21	10.4%	0.71	7.03	10–174	10–114
Lion 2	14.9	−0.09	10.2%	0.68	9.05	13–595	13–180
Bengal Tiger	11.1	0.026	7.1%	0.82	7.2	13–219	10–110
Siberian Tiger	10.7	−0.11	11.0%	0.43	10.36	14–838	10–225
Sumatran Tiger	28.5	−0.18	9.3%	1.03	10.7	21–2393	12–430

E, Young's modulus (in kPa) between the linear strain limit and 50% strain. F_0_, fundamental frequency (in Hz) implementing the linear and exponential model in Eq. 1.

### Rheometry measurements

**Figure 8:**
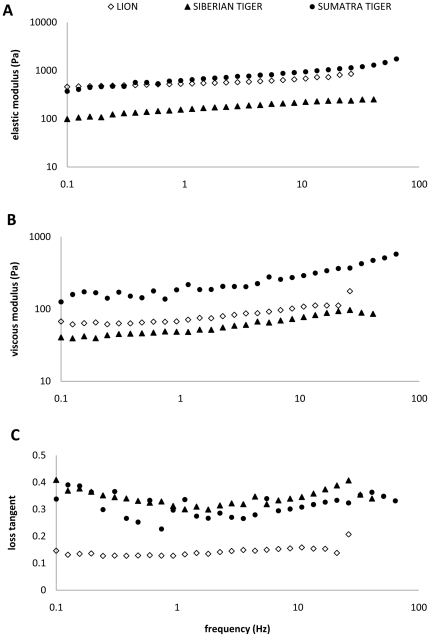
Elastic and viscous moduli of lion and tiger vocal fold tissues increase with frequency, where elastic modulus dominates material response to oscillation (i.e., loss tangent values are 0.1–0.4). Shear elastic modulus *G*′ (**A**), viscous modulus *G*″ (**B**) and loss tangent = *G*″/*G*′ (**C**) lion, Siberian tiger, and Sumatran tiger vocal fold tissue as a function of frequency.

**Figure 9:**
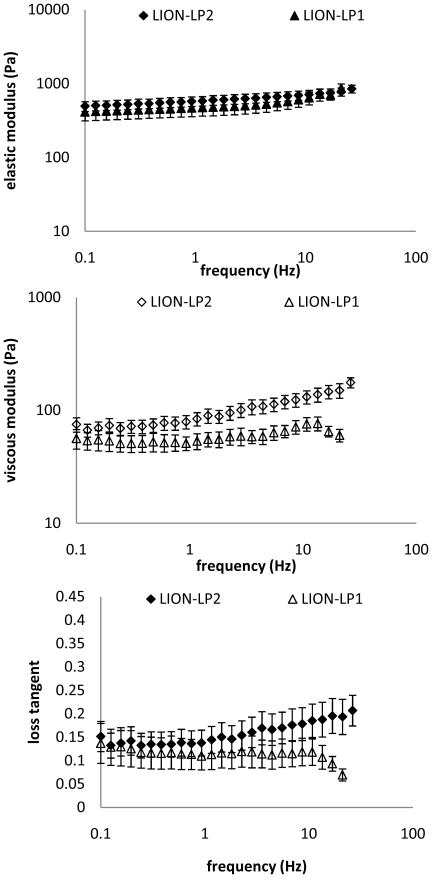
Viscous modulus and loss tangent diverge at frequencies larger 10 Hz indicating different behavior between the fat-free and fat-containing layer. Mean elastic modulus *G*′ (**A**), viscous modulus *G*″ (**B**), and loss tangent = *G*″/*G*′ (**C**) of the lion LP_2_ and LP_1_ as a function of frequency. Error bars correspond to ±1 standard error(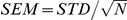).

Elastic shear modulus *G*′ and viscous shear modulus *G*″ ranged between 100–2000 Pa and 40–600 Pa, respectively, across frequency for three species (Figure 8). The elastic modulus dominated the viscous properties of the tissues. This was evident by calculating the loss tangent *G*″/*G*′, where all values were less than 1 (0.33±0.04 across all frequencies for Sumatran and Siberian tiger tissues; 0.15±0.02 for lion tissues). Figure 9 shows elastic and viscous moduli for the two regions of lamina propria of the lion. Most dramatically the loss tangent modulus of the two layer diverges beyond frequencies of 10 Hz. Unfortunately we were only able to test frequencies up to 20 Hz.

### Predicted fundamental frequency

Stress-strain data were incorporated into the ideal string formula (Eq. 5). The combination of linear and exponential stress-strain models and vocal fold length for each specimen was used in the formula. Between the original vocal fold length and 60% elongation, the string model predicted a minimum fundamental frequency of about 10 Hz and a maximum fundamental frequency ranging between 110 and 430 Hz across 5 individuals.

### Predicted phonation threshold pressure

**Figure 10:**
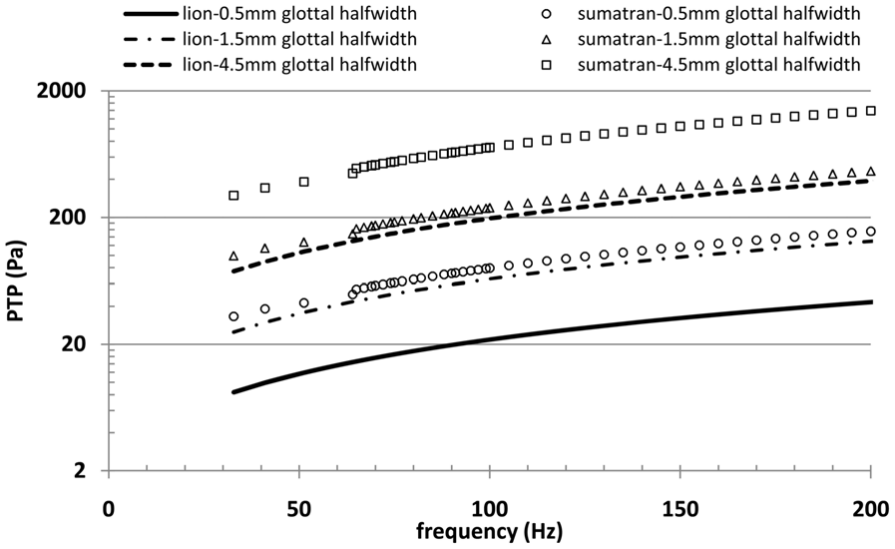
Phonation threshold is surprisingly low for Panthera vocal folds. The values were calculated from biomechanical properties of lion and Sumatran tiger across frequency with varying glottal half-widths.

Phonation threshold pressure (P_th_) predictions using Eq. 6 were 5–2000 Pa, depending on glottal half-width assumed for adduction and the fundamental frequency. If glottal half width is no more than 0.15 mm the range of P_th_ predictions was 8.3–130 Pa for lion and 33–460 Pa for Sumatran tiger at F0 values of 33–200 Hz. When accounting for lower F0 values of *Panthera*, 30–90 Hz, maximum predicted P_th_ dropped to 59 and 220 Pa, respectively (Figure 10). Each curve fits to a linear function with R^2^ values of 0.9996 or 0.9997.

## Discussion

The results of our investigation provide four new findings that contribute to the understanding of vocal fold functional morphology and the role of fat in vocal folds.

First, vocal folds of *Panthera* demonstrated a highly organized structure. There is a deep (lateral) layer of *lamina propria* that contains adipocytes embedded in collagen and fewer elastin fibers. The more superficial (medial or lumen-side) layer is fat free and characterized by dense and compact extracellular matrix. Hyaluronan was identified in both layers. There are finger-like projections and indentations at the interface between the *lamina propria* and the epithelium, similar to what is found in the esophagus or the *rete ridge* arrangement between dermis and epidermis [38]. The larger surface area between epithelium and the connective tissue beneath improves efficiency of nutrient, oxygen, and waste exchange. It also increases the number of physical binding sites connecting the epithelium to the *lamina propria* so that the tissue could withstand greater forces than for a simpler smooth interface.

Second, Young's modulus ranged between 10 kPa and more than 2 MPa. The shape of the stress-strain curve is linear in the low-strain region (at strains between 0 and 11%) and exponential above the linear strain limit of 11%. This stress-strain behavior resembles that of vocal folds from other species qualitatively, but is quantitatively different. For a strain of 40%, lion and tiger vocal folds show a lower Young's modulus than humans or rhesus monkeys (Table 3). This confirms earlier findings that vocal fold viscoelastic properties contribute to vocal differences at the individual, sex and species level (e.g. [2], [3], [5], [6], [39]). All animals in this study were of old age and were outside the mating season at the time of death. Factors such as age or season (mating versus non-mating) have not been tested, but are likely to be reflected in viscoelasticity of vocal folds.

**Table 3:** Mean tangent Young's moduli of lamina propria tissue of vocal folds from males of different mammal species.

Species	vocal fold length in vivo	Young's modulus	source data
rat (*Rattus norvegicus*)	1 mm	<100 kPa	Riede et al. 2011
rhesus monkey (*Macaca mulatta*)	9 mm	10369 kPa	Riede 2010
human (*Homo sapiens*)	15 mm	≈2000 kPa	Chan et al. 2007
mule deer (*Odocoileus hemionus*)	26 mm	819 kPa	Riede et al. 2010
Rocky Mountain elk (*Cervus elaphus nelsoni*)	32 mm	235 kPa	Riede & Titze 2008
Sibirian tiger (*Panthera tigris*)	40 mm	399 kPa	this study

Provided here is a single value for Young's modulus at 40% strain. At large strains the stress-strain curve of the lamina propria is exponential and therefore the Young's modulus is continuously changing.

Third, the elastic shear modulus (*G*′) ranged between 0.1 and about 2 kPa, and the viscous shear modulus (*G*″) between 0.05 and about 0.5 kPa. The value 0.2 kPa has been reported for diced and rinsed subcutaneous fat [34] and 40 kPa for non-minced subcutaneous fat [40]. Apparently the moduli for lion and tiger lamina propria ranges at the lower end for fat-containing tissue. Interestingly we found differences between the fat-free and fat-containing layer of *lamina propria*. We expected that the fat laden, lateral region of the *lamina propria* (LP1) would show lower viscoelastic moduli owing to the compactness of fibrillar proteins in LP2. Data indicate that loss tangent for the two layers diverges beyond 10 Hz. Unfortunately we were not able to extend the frequency range of the rheometry closer to phonation relevant frequencies, and therefore this observation remains to be further investigated.

Fourth, F0 range and P_th_ can be predicted from viscoelastic properties. The string model suggests F0 between 20 and 400 Hz. This is in agreement with F0 ranges measured in natural vocalizations [16], [17], [41]–[44]. For example, a lion's roar shows F0 between 40 and 200 Hz [16]–[18], a tiger's long distance advertisement call has a F0 of about 160 Hz [19]. Phonation threshold pressure was predicted at about 0.3 kPa which is in agreement with findings in excised larynx experiments in tiger (0.2–0.4 kPa) [9]. This value is surprisingly lower than in normal human conversational speech. Low shear elastic and viscous properties of *Panthera* vocal folds help to ease phonation onset. These findings support the hypothesis that morphology and vocal function of vocal folds are linked. F0 and P_th_ can be predicted from morphological information.

Finally, what role does fat play in a specialized vocal fold, like that of tigers and lions? In the following section we discuss two new (not mutually exclusive) hypotheses: a) fat eases phonation by optimizing vocal fold shape and b) fat possesses a protective function.

### The function of fat in the vocal fold

Hast [10] suggested that fat increases a vocal fold's mass and thereby helps to produce low fundamental frequencies. However, a robust relation between F0 and vocal fold mass does not exist [28], and very large vocal folds can be involved in very high oscillation frequencies (e.g. [45]). It is the effective mass in motion that determines F0, rather than the total mass of the vocal fold. Adipose tissue is a form of connective tissue. It has amorphous characteristics rather than a fibrous construction like collagen or elastin. Fat density (0.85 g/cm^3^) is lower than that of collagen or elastin (1.05 g/cm^3^). The fat certainly contributes to volume but its lower density causes a less than proportional increase in mass. The deep position inside the lamina propria suggests that it is not contributing to the effective mass in vibration. Only the more superficial fat-free layer becomes involved in tissue oscillation because of its close proximity to aerodynamic forces and the different viscoelastic properties of the two *lamina propria* layers. Rather than adding mass, we believe that fat helps to shape the vocal fold geometry. The thick and flattened medial surface of the *Panthera* vocal fold, giving the vocal folds a rectangular shape, contributes to lower P_th_. The thick and very compact medial vocal fold layer in combination with a large medial surface help to produce low fundamental frequencies and very high sound intensities [13].

Fat is not often found in vocal folds. If it occurs, it is located deep in the *lamina propria*, as in rhesus monkey vocal folds (*Macaca mulatta*) [6]. The deep position in the vocal fold is probably the only location where adipocytes can survive. They probably would not survive extreme shear stresses. In both, Panthera and rhesus monkeys, high amplitude sounds associated with extreme tissue vibration (screams in rhesus monkeys and roars in lions and tigers) are normal aspects of the vocal repertoire.

Secondly, we suggest that fat also provides a protective function. Its deep position could serve a cushioning function for overlaying tissue. Fat is more deformable than densely packed fibro-elastic tissue and therefore can absorb large lateral deformations. Fat is widely found as a cushion material to absorb compression stress (e.g. in the sole, the knee or in the fundament/buttocks). We observed that the deep fatty layer contains relatively large blood vessels, many more than the fat-free layer of the *lamina propria*. Fluid and blood circulation is in general a problem in oscillating material because blood vessels are sensitive to severe bending or shear, which cause rupture. The embedding of vessels in a layer of fat seems to be a strategy to secure vessel intactness because any deformation will be smoothed by the large fat cells. Furthermore, fat accumulation in connective tissue has been identified as a source for repair mediated by stem cells. Adipocytes can be a source of mesenchymal stem cells [46]. Cells derived from cultured adipose tissue of patients who were being treated for glottic insufficiency, exhibited markers of mesenchymal stem cells. It was possible to differentiate those cells among others into adipogenic lineages [47]. Given that the roaring vocalization in lions and tigers may cause tissue damage due to large tissue oscillations, a reservoir of cells for fast tissue repair would guarantee stable phonation and therefore provide an advantage.
